# The letter to Editor regarding ‘Patient-reported outcomes of esthetics, function and oral hygiene with single dental implants 10–15 years after placement: a cross-sectional study’

**DOI:** 10.2340/aos.v84.45202

**Published:** 2025-12-22

**Authors:** Jiayi Chen

**Affiliations:** Suzhou Wujiang District Hospital of Traditional Chinese Medicine, Suzhou City, PR China

Dear Editor,

The recent study by Bengtsson et al. provides valuable long-term data on patient-reported and clinician-assessed outcomes of single dental implants after 10–15 years [1]. The authors are commended for addressing an important research gap by integrating both subjective and objective evaluations, which align with current recommendations from the VIII European Workshop on Periodontology emphasizing patient-reported outcome measures (PROMs) in implant dentistry.

The findings of this study demonstrate high patient satisfaction with function and esthetics (mean visual analog scales (VAS) 8.6–9.9), confirming the durability of single-implant restorations over time. However, the lower scores observed for peri-implant mucosal esthetics (mean pink esthetic score (PES) 6.2 ± 1.4) merit attention. This result suggests that even when crowns maintain favorable color and form, soft-tissue dynamics may compromise the overall esthetic harmony. These findings echo the conclusions of Belser et al., who also highlighted mucosal factors as key determinants of esthetic success [2].

The study appropriately acknowledges the limitation of using non-validated questionnaires. Incorporating standardized PROM instruments in future studies would enhance comparability across populations and time points. Additionally, given the high prevalence of peri-implant mucositis (88.9%), longitudinal data on maintenance frequency and hygiene compliance would be valuable for clarifying the relationship between supportive care and long-term tissue stability.

Overall, Bengtsson et al. contribute meaningful evidence to the field by emphasizing both the functional longevity and esthetic perception of implants after more than a decade of service. Their work reinforces the necessity of combining biological, esthetic, and patient-centered metrics when evaluating long-term implant success.

## References

[1] Wallin Bengtsson V, Lindahl C, Scholander S. Patient-reported outcomes of esthetics, function and oral hygiene with single dental implants 10–15 years after placement: a cross-sectional study. Acta Odontol Scand. 2025 Jan 21;84:47–53. 10.2340/aos.v84.42724 39835673 PMC11806213

[2] Belser UC, Grütter L, Vailati F, Bornstein MM, Weber HP, Buser D. Outcome evaluation of early placed maxillary anterior single-tooth implants using objective esthetic criteria: a cross-sectional, retrospective study in 45 patients with a 2- to 4-year follow-up using pink and white esthetic scores. J Periodontol. 2009 Jan;80(1):140–51. 10.1902/jop.2009.080435 19228100

